# Preparation of high-capacity magnetic polystyrene sulfonate sodium material based on SI-ATRP method and its adsorption property research for sulfonamide antibiotics

**DOI:** 10.1186/s13065-019-0658-8

**Published:** 2020-01-14

**Authors:** Huachun Liu, Bolin Gong, Yanqiang Zhou, Zhian Sun, Xiaoxiao Wang, Shanwen Zhao

**Affiliations:** 1 School of Chemistry and Chemical Engineering, North Minzu University, Yinchuan, 750021 People’s Republic of China; 2 No. 204 Wenchang North Street, Xixia District, Yinchuan, People’s Republic of China

**Keywords:** Polystyrene sulfonate sodium (PSS) magnetic material, Surface-initiated atom-transfer radical polymerization (SI-ATRP), Sulfonamide antibiotic, Adsorption performance, High performance liquid chromatography (HPLC)

## Abstract

A novel polystyrene sulfonate sodium (PSS) magnetic material was prepared by surface-initiated atom transfer radical polymerization (SI-ATRP). The starting materials were brominated magnetic material as the carrier and macroinitiator, sodium styrene sulfonate (NaSS) as the monomer, and cuprous bromide/2,2′-dipyridyl as the catalyst system. The PSS material was characterized by Fourier transform infrared spectroscopy (FT-IR), elemental analysis, transmission electron microscope (TEM), thermogravimetric analysis (TGA), scanning electron microscopy (SEM), and a vibrating sample magnetometer (VSM). The adsorption properties of the material were then investigated on sulfa antibiotics. The kinetic and thermodynamic parameters were determined in adsorption of sulfamethazine (the smallest molecular-weight sulfonamide). The adsorption amount of sulfamerazine free acid (SMR) was found to increase with the initial concentration and temperature of SMR in solution. The adsorption effect was maximized at an initial concentration of 0.6 mmol/L. The static saturation adsorption capacity of the material was 33.53 mg/g, Langmuir and Freundlich equations exhibited good fit. The thermodynamic equilibrium equation is calculated as ΔG < 0, ΔH = 38.29 kJ/mol, ΔS > 0, which proves that the adsorption process is a process of spontaneous, endothermic and entropy increase. Kinetic studies show that the quasi-second-order kinetic equation can better fit the kinetic experimental results, which is consistent with the quasi-second-order kinetic model. The experimental results of kinetic studies were well fitted to a quasi-second-order kinetic equation. High performance liquid chromatography (HPLC) of an actual milk sample treated by the PSS magnetic material confirmed the strong adsorption of SMR from milk.
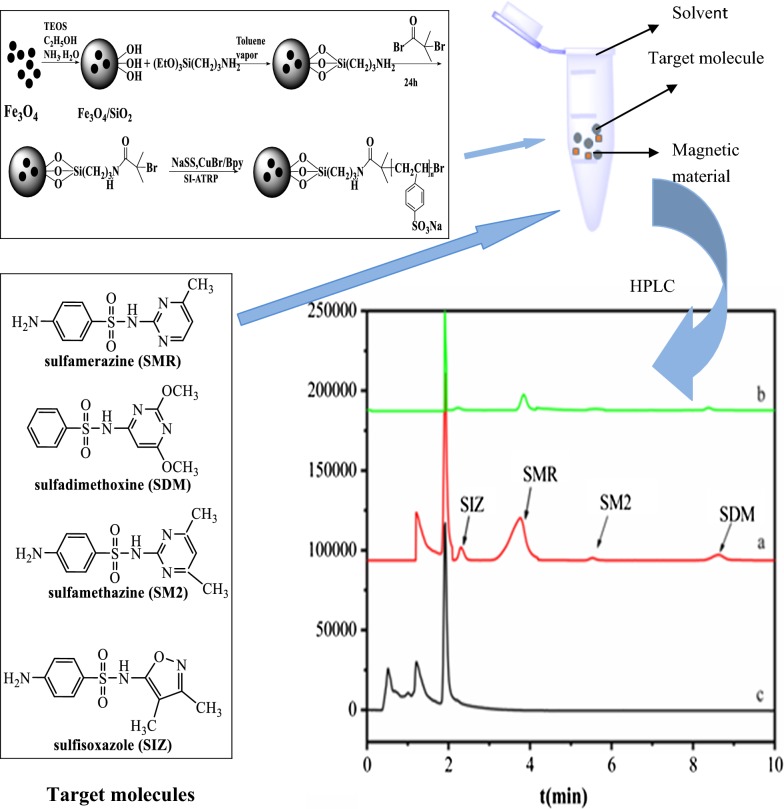

## Introduction

Sulfa drugs (SAs) are a class of synthetic anti-infective drugs with a wide antibacterial spectrum. They are also convenient to use and stable in nature. Owing to these advantages, SAs are widely used in aquaculture and animal breeding [1–4]. However, bacteria easily become resistant to sulfa drugs, and sulfa drug residues can accumulate in animals after long-term use. Therefore, the United Nations Codex Alimentarius Commission (CAC) and many national regulations have limited the total amount of SAs in animal feed to 0.11 mg/kg [5, 6]. At present, sulfa drugs in China are mainly treated by simple physicochemical methods [7, 8], SBR (sequencing batch activated sludge leads to normal flora imbalance in the body [9]), and adsorption methods [10, 11].

Surface-initiated atom-transfer radical polymerization (SI-ATRP) is a new actively controlled polymerization technology that enables “active” polymerization. Because it controls the graft chain length [12–14], SI-ATRP grafting is a popular surface graft-modification technique for various materials. Using SI-ATRP technology, Niu et al. [15] obtained an aminated resin with higher adsorption capacity for Cu(II), Pb(II), Cr(VI), and As(V) than traditional resins. By the same technology, Chen et al. [16] prepared a chelate resin with a 4-vinylpyridine ring as the functional group. This resin readily adsorbs Cr(VI), Pb(II), and Cr(III).

The unique magnetic properties of Fe_3_O_4_ magnetic nanomaterials have been widely exploited in magnetic fluids, data storage, and pollutant treatments [17, 18]. Jin et al. [19] prepared monodispersed carboxylated Fe_3_O_4_ magnetic nanoparticles, and Cheng et al. [20] studied the adsorption performance of amino-functionalized mesoporous magnetic nanoparticles on Cu(II) in water, but not in actual samples. Therefore, the performance of their nanoparticles in real applications is unknown. To fill these gaps, we prepared magnetic materials by grafting modified Fe_3_O_4_ magnetic nanoparticles onto sodium styrene sulfonate, and testing their ability to adsorb antibiotics from food. To this end, we detected the adsorbed and remnant sulfa antibiotics in a food source (milk) treated by the magnetic material, which has not been reported in the prior literature.

In this study, the carrier/initiator was a brominated magnetic material, the monomer was sodium styrene sulfonate (NaSS), and the catalyst was cuprous bromide/2,2′-bipyridyl. A novel sodium polystyrene sulfonate magnetic material was prepared by the SI-ATRP technique. Adsorption and removal experiments of the sulfa antibiotics were performed under various conditions of the magnetic material, yielding informative results.

## Materials and methods

### Apparatus

Experiments were carried out in the following instruments: an LC-20AT high performance liquid chromatograph (Shimadzu Corporation, Japan), a JEM-2100 transmission electron microscope (JEM, Japan), a JJ-1 precision factory electric mixer (Shanghai Specimen Model Factory), a collecting thermostatic heating magnetic stirrer (Zhengzhou Changcheng Branch Industry and Trade Co., Ltd.), a KQ-3200E ultrasonic cleaner (Kunshan Ultrasonic Instrument Co., Ltd.), a BS-224S electronic balance (Sedolis Scientific Instrument Co., Ltd.), an SHZ-C type water bath constant-temperature oscillator (Shanghai Pudong Physical Optics Instrument Factory), a TU-1810 UV–visible spectrophotometer, (Beijing Pu Analysis General Instrument Co., Ltd.), a TGL-20 M high-speed desktop centrifuge (Changsha Xiangyi Centrifuge Co., Ltd.) and a Fourier transform infrared spectrometer (Shimadzu, Japan). The absorbance was measured by the TU-1810 UV–Vis spectrophotometer purchased from Beijing Pu Analysis General Instrument Co., Ltd. The supernatant after adsorption by the material was photometrically determined to determine the absorption wavelength of the sulfonamides. Then, spectral scanning was performed, and different absorbances were measured and processed by UVWin5 software to complete the experiment. The actual sample was analyzed by LC-20AT high performance liquid chromatography (Shimadzu Corporation, Japan). The instrument was equipped with DGU-20A3 degasser, 2 LC-20AT solvent transfer pumps (divided into A and B pumps), and 7725i manual feed. Sampler, CTO-20A column oven, SPD-20A UV–Vis detector and CBM-20A system controller. Diamonsil C18 column (150 mm × 4.6 mm, 5 μm), mobile phase acetonitrile–water (25:75, v/v) and filtered through a 0.45 μm filter with a flow rate of 0.8 mL/min and a detection wavelength of 270 nm and set the injection volume of 20 μL.

### Reagents and materials

Sodium styrene sulfonate (NaSS), sulfamerazine free acid (SMR), sulfadimethoxine (SDM), sulfafurazole (SIZ), sulfadimidine (SM2), *N*,*N*-dimethylformamide (DMF), 3-aminopropyltriethoxysilane (MSDS), α-bromoisobutyryl bromide,hydroxylamine hydrochloride, oleic acid, tetraethyl orthosilicate (TEOS), cuprous bromide (CuBr) and 2,2′-bipyridine (Bpy) were purchased from Aladdin Reagent Co., Ltd. (Shanghai, China). Ferric chloride hexahydrate (FeCl_3_·6H_2_O), ethylenediaminetetraacetic acid (EDTA), aqueous ammonia (NH_3_·H_2_O), hydrochloric acid (HCl), acetonitrile,methylbenzene,sodium hydroxide (NaOH), absolute ethyl alcohol,tetrahydrofuran, and triethylamine were purchased from Damao Chemical Reagent Factory (Tianjin, China).

### Preparation of magnetic Fe_3_O_4_/SiO_2_ nanocomposite particles

FeCl_3_·6H_2_O (60 mL, 0.05 mol/L) and ethanol–water (1:1 v/v) were placed in a round-bottomed flask and heated to 50 °C with magnetic stirring. At the start of stirring, 0.0511 g hydroxylamine hydrochloride was quickly added to the mixture. After 5 min of stirring, the pH was adjusted to > 9.0 by adding 25% ammonium hydroxide. Next, 1 mL oleic acid was slowly (dropwise) added to the solution while warming to 70 °C for 10 min. After stirring for a further 30 min at 70 °C, the solution was allowed to cool to room temperature. The solids were then separated by a solid magnetic field. The resulting black precipitate was washed several times with absolute ethanol and vacuum-dried at 60 °C.

Weighed Fe_3_O_4_ particles (1.00 g) were ultrasonically dispersed in 100 mL ethanol–water (4:1 v/v) for 10 min. The dispersed solution was transferred to a 250-mL three-necked bottle. After adding 2 mL 25% ammonium hydroxide and (slowly) 1 mL TEOS, the mixture was mechanically stirred until uniform, and the reaction was sealed for 24 h. After completion of the reaction, the solution was repeatedly washed with distilled water under the magnetic-field separation conditions until it became neutral and no longer cloudy.

### Synthesis of Fe_3_O_4_/SiO_2_ grafted PSS composites

Dried Fe_3_O_4_/SiO_2_ (1.00 g) solid particles were weighed into a 100 mL three-necked flask. After adding 20 mL of absolute ethanol, the particles were ultrasonically dispersed for 15 min. When the dispersion was complete, 3 mL of MSDS was added and the reaction was heated in a 90 °C oil bath for 24 h After completion of the reaction, the mixture was washed successively with toluene, secondary water and absolute ethanol until neutral, and vacuum-dried at 60 °C.

The aminosilylated Fe_3_O_4_/SiO_2_ (0.5 g) was dispersed in 30 mL of tetrahydrofuran, and the reaction was stirred for 30 min in an ice bath. Triethylamine (1.25 mL) was then added dropwise, and the mixture was stirred at room temperature for 10 min. After dropwise of 1 mL α-bromoisobutyryl bromide, the reaction was left at room temperature for 20 h to complete the reaction. The product was washed twice with tetrahydrofuran, distilled water and acetone, and vacuum-dried at 60 °C.

Initiator-modified Fe_3_O_4_/SiO_2_ (0.3 g) was weighed into a 50 mL round-bottomed flask. After adding 0.0743 g Bpy, 0.0213 g CuBr, and 0.995 g sodium styrenesulfonate in 40 mLNN-dimethylformamide–water solution, the Fe_3_O_4_/SiO_2_ particles were ultrasonically dispersed for 15 min. Nitrogen was then deaerated for 30 min at room temperature, and the reaction was sealed at 60 °C for 20 h. After the reaction, the polymerization product was separated by a magnetic field, and the impurities in the precipitate were removed by sequential washing with saturated EDTA, distilled water and acetone (two washes in each cleaning agent). The product, polystyrene sulfonate sodium (PSS) magnetic material, was vacuum-dried at 60 °C.

### Adsorption experiments

Saturated adsorption capacity experiment: PSS magnetic material (0.1 g) was weighed into a 50 mL erlenmeyer flask. After adding 0.1 mol of 10 mL SMR to acetonitrile–NaOH solution (9:1, v/v) and shaking for 24 h in a water bath thermostat, the supernatant was extracted. The remaining concentration of SMR in the supernatant was determined, and the adsorbed amount (mg/g) was calculated as follows [21]:

1 $${\text{Q}} = \frac{{\left( {C_{0} - C_{e} } \right)V}}{m}$$

where Q is the amount of adsorption (mg/g), C_0_ and C_e_ are the initial and adsorption equilibrium concentrations of SMR, respectively (mmol/L), *V* is the solution volume (mL), and *m* is the mass (g) of the PSS magnetic material.

Adsorption selectivity: To determine the adsorption selectivity of SMR, we prepared additional target molecules SDM, SM2, and SIZ, which are similar to SMR. Into solutions of 0.6 mmol/L acetonitrile (10 mL) and 0.1 mol/L NaOH (9:1 v/v) was weighed 0.1 g of magnetic material. The mixtures were oscillated in a water bath at constant temperature. After static adsorption for 24 h, the absorbances of the supernatants were measured in a UV–visible spectrophotometer, and the adsorption amounts of the magnetic materials were calculated by Eq. (1).

Adsorption kinetics: The adsorption kinetics were measured under the condition of pH > 7. Magnetic material was added to the same concentration of SMR solution. The mixture was continually oscillated in a constant-temperature oscillator and sampled regularly. The adsorption amounts were determined from the absorbances measured at each sampling time, and an adsorption amount–time curve was plotted to determine the adsorption rate. The experimental results were analyzed by different kinetic models and the kinetic reaction order was determined.

Adsorption thermodynamics: The adsorption thermodynamics were measured under the condition of pH > 7, a constant amount of the magnetic materials was added to different initial concentrations of SMR solution. The solutions were continually oscillated in a constant-temperature oscillator. The adsorption was balanced and sampled. The adsorption isotherm was obtained by plotting the equilibrium concentrations and the corresponding equilibrium adsorption amounts as the abscissa and ordinate, respectively. The adsorption amounts were investigated at different temperatures, and the relevant thermodynamic parameters were calculated from the results.

### Adsorption properties under different pH

0.1 g of sodium polystyrene sulfonate magnetic material was placed in an aqueous solution at 25 °C, and the pH values were 4.0, 5.0, 6.0, 7.0, 8.0, 9.0, and 10.0, respectively. The initial concentration of the SMR solution was 0.6 mmol/L. Adsorption was carried out for 7.5 h under magnetic stirring. And calculate the adsorption amount of SMR.

### Sample processing

Milk samples (5 mL) were accurately transferred into a 50 mL centrifuge tube. After adding a certain amount of the sulfa drug standard solution, 1 mL hydrochloric acid solution (1 mol/L) and 15 mL acetonitrile, the mixture was ultrasonicated for 20 min, then centrifuged at 4000 rpm for 10 min. The supernatant was collected through a filtration membrane, spin-dried, then reconstituted in 5 mL of acetonitrile. The vials were placed in the refrigerator for later use.

## Results and discussion

### Preparation of magnetic PSS

**Fig. 1:**
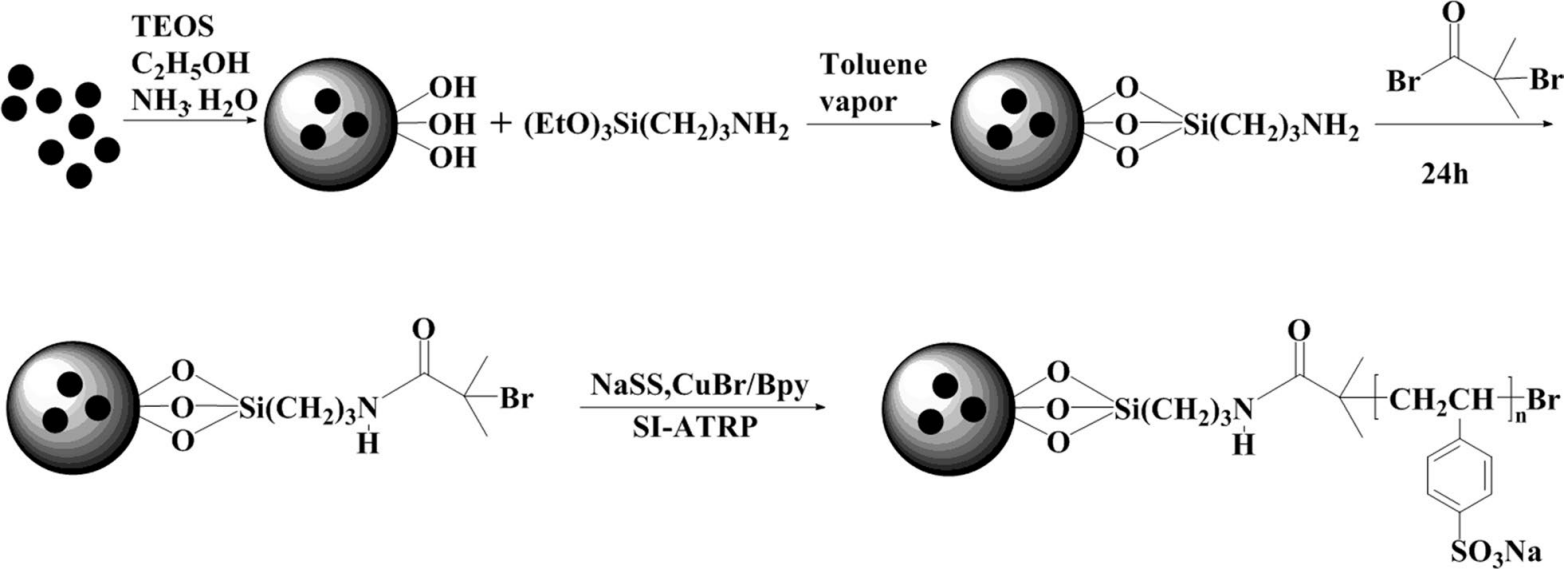
Synthesis of PSS magnetic materials

Magnetic Fe_3_O_4_ nanoparticles were prepared by the coprecipitation method. Their surfaces were then coated with SiO_2_ to form a core–shell structure. The coated nanoparticles were sequentially reacted with 3-aminopropyltriethoxysilane and α-bromoisobutyryl bromide to obtain the SI-ATRP initiator. Next, the polymerization monomer NaSS was grafted onto the initiator surface by SI-ATRP polymerization in an acetonitrile–NaOH solvent medium. The reaction was catalyzed by CuBr and Bpy was the complexing agent. The preparation process of the magnetic PSS adsorption material is shown in Fig. 1.

### Instrumental characterization

**Fig. 2:**
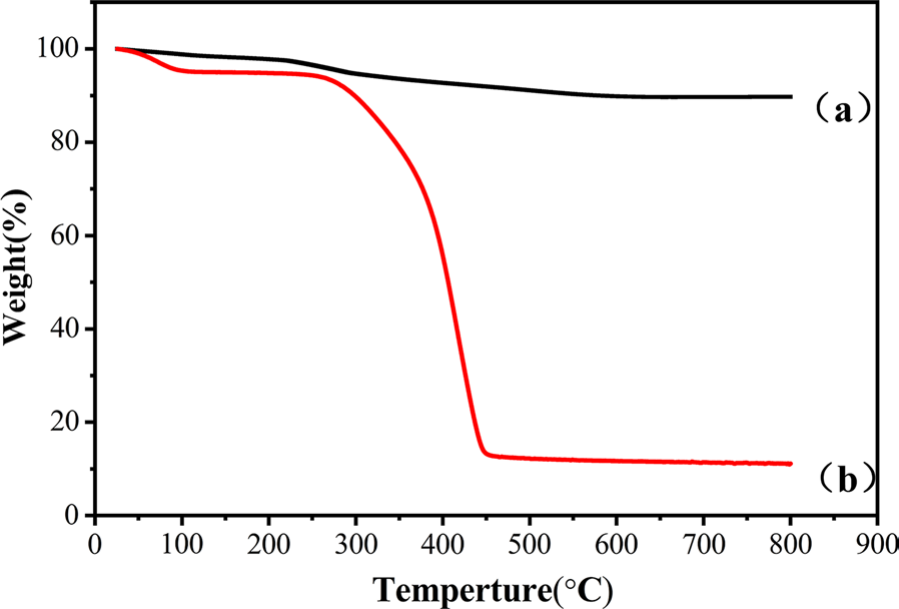
TGA curves of the Fe_3_O_4_/SiO_2_/Br (**a**) and PSS (**b**) magnetic microspheres

Figure 2 displays the thermogravimetric plots of Fe_3_O_4_/SiO_2_/Br and PSS. The Fe_3_O_4_/SiO_2_/Br(a) was highly stable, losing only 10% of its weight between room temperature and 800 °C. Below 150 °C, the weight loss of Fe_3_O_4_/SiO_2_/Br and PSS is mainly attributable to evaporation of the residual ethanol layer. As the temperature was raised from 300 to 500 °C, the weight loss from PSS was large and rapid. At 500 °C, the PSS had lost 80.3% of its initial weight, mainly by decomposition of NaSS.

The SI-ATRP initiators before and after the NaSS grafting were characterized by elemental analysis. The C and H, S contents were higher in the final SI-ATRP-based adsorbent than in the SI-ATRP initiator (Table 1). The graft amount was calculated as follows [22]:

2 $${\text{Graft}}\;{\text{amount}} = {{M_{n} W_{\text{S}} } \mathord{\left/ {\vphantom {{M_{n} W_{\text{S}} } {N_{\text{S}} M_{\text{S}} }}} \right. \kern-0pt} {N_{\text{S}} M_{\text{S}} }}$$

where W_S_ is the percentage of the S element per unit volume of the magnetic material surface; N_S_ is the number of S elements per unit volume of the monomer; M_S_ is the relative molecular mass of the S element; M_n_ is the molar mass of the monomer. The graft amount calculated by Eq. (2) was 27.99 μmol/m^2^, indicating that the NaSS had been successfully grafted onto the surface of the SI-ATRP initiator.

**Table 1:** Elemental analysis results

Sample	C	H	N	S
Fe_3_O_4_/SiO_2_/NH_2_/Br	83.51	5.705	1.013	–
PSS	84.90	5.975	3.272	1.327

**Fig. 3:**
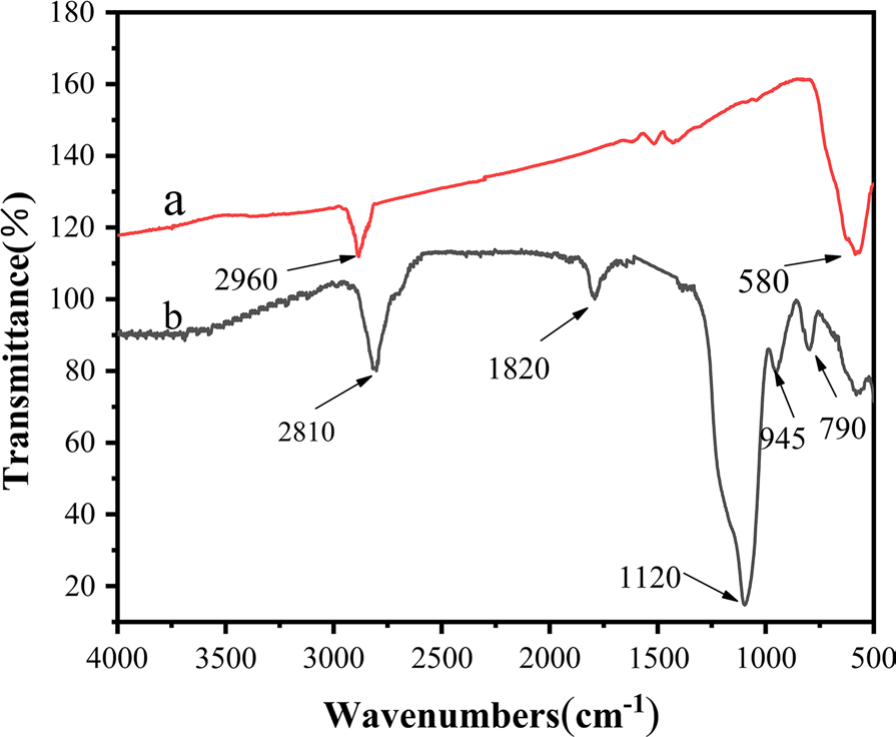
Infrared spectra of Fe_3_O_4_ (**a**) and PSS (**b**)

Figure 3 shows the infrared spectra of the Fe_3_O_4_ and PSS materials. In the spectrum of oleic acid modified Fe_3_O_4_, the peaks around 2960/cm and 580/cm are the characteristic absorption peaks of –CH_3_ in oleic acid and the stretching vibration of Fe–O bonds, respectively. In the PSS spectrum, the strong absorption peak at 1120/cm is attributable to asymmetric stretching vibrations of Si–O–Si. Peaks attributable to Si–O vibrations (790/cm) and Si–O–H vibrations (945/cm) are also clarified. These peaks indicate that SiO_2_ was successfully coated on the Fe_3_O_4_ surface. The in-plane skeleton vibration of the benzene ring at 1450/cm and the characteristic peak of the Fe–O bond at 580/cm are also less affected by the benzene ring. The absorption peak at 2810/cm is assigned to stretching vibrations of saturated C–H. The characteristic C–C peak is absent. The stretching vibration peak of C=O at 1820/cm indicates that the successful preparation of PSS magnetic material.

**Fig. 4:**
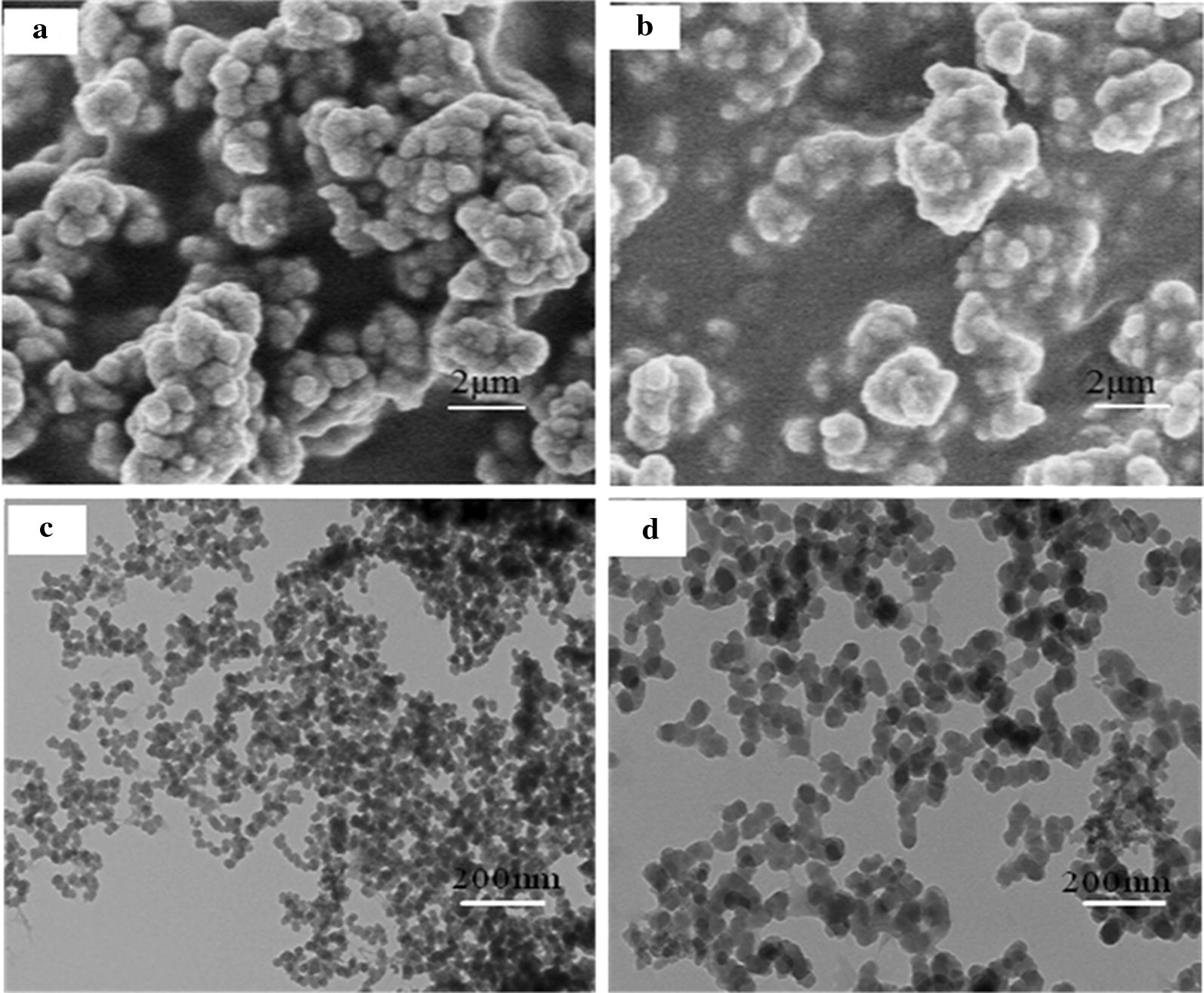
SEM images of the PSS magnetic material before (**a**) and after (**b**) SMR adsorption and TEM of PSS material

Scanning electron microscopy was used to characterize the surface morphology and structure of PSS materials before and after adsorption. The results are shown in Fig. 4, in which Fig. 4a is a magnetic material before adsorption, and Fig. 4b is a saturated magnetic material after adsorption of SMR. It can be seen in the figure that the surface morphology of the resin before and after adsorption has undergone a great change. Before the adsorption, the material morphology is obvious and pores with different sizes are formed, and the surface is uneven. The surface of the resin became smooth after adsorption and the pore size and size also changed. It shows that after the adsorption of SMR, the surface of the material changes significantly, so it shows that the magnetic material has good adsorption to SMR. At the same time, the synthesized materials were characterized by transmission electron microscope (TEM) [23]. The results are shown in Fig. 4c, d. It can be clearly seen in the figure that the synthesized material has a core–shell structure, which proves that the magnetic material is successfully prepared.

**Fig. 5:**
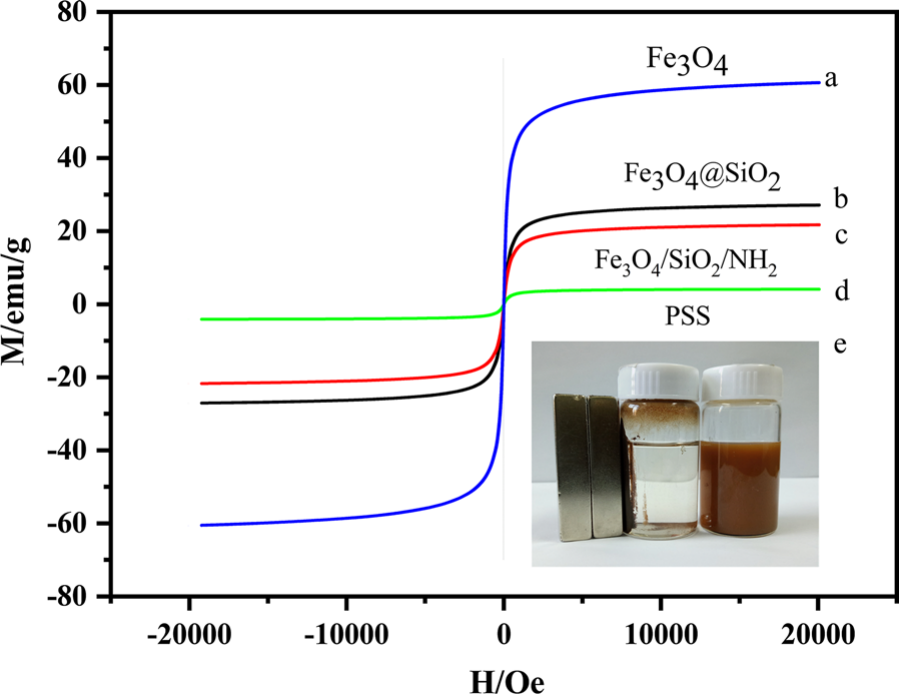
Magnetization characteristics (hysteresis curves) of **a** Fe_3_O_4_ nanomaterials, **b** Fe_3_O_4_/SiO_2_, **c** Fe_3_O_4_/SiO_2_/NH_2_, **d** PSS. **e** Photograph of magnetic separation in PSS

The oleic acid-modified Fe_3_O_4_ nanoparticles, Fe_3_O_4_/SiO_2_, Fe_3_O_4_/SiO_2_/NH_2_ and PSS were analyzed by VSM at room temperature. The magnetization curves of the four materials are presented in Fig. 5. The inset is a photograph of the magnetic separation under an external magnetic field. The saturation magnetization of the Fe_3_O_4_ nanoparticles was 60.67 emu/g, close to the reported magnetic susceptibility of this material. The measured value is smaller than the saturation magnetic susceptibility theoretically obtained from the standard material, possibly because of particle surface effects. After each step, the saturation magnetization of the PSS nanoparticles was reduced to 10.14 emu/g and higher than the literature report [24, 25], because the magnetic responsiveness of the PSS was suppressed by the non-magnetic layer coated on its surface. Before the magnetic field was applied, the PSS were uniformly dispersed in the acetonitrile solution (Fig. 5e), but under the external magnetic field, the tan particles were rapidly attracted to the wall of the bottle. On the side, the solution became transparent (Fig. 5e, left), confirming the high magnetic responsiveness of the PSS and its suitability as a magnetic separation carrier.

### Adsorption performance of PSS magnetic materials

**Fig. 6:**
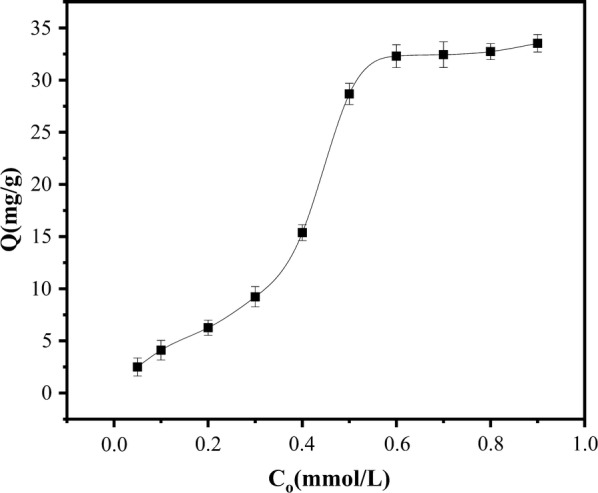
Effect of initial SMR concentration on the adsorption capacity of PSS magnetic material

Effect of initial SMR concentration on adsorption capacity: Concentration is an important factor affecting the adsorption process. Figure 6 plots the SMR adsorption capacity of the PSS magnetic material versus the initial SMR concentration. The amount of adsorbed SMR gradually increased with initial concentration, likely because the probability of contact between SMR and adsorbent increases when the absorbent is dense in the solution. When the initial concentration exceeded 0.6 mmol/L, the adsorption amount saturated and was not further changed by increasing the initial SMR concentration. The adsorption amount was 33.53 mg/g, higher than the reported value [26, 27]. Therefore, 0.6 mmol/L was deemed the optimal initial SMR concentration.

**Fig. 7:**
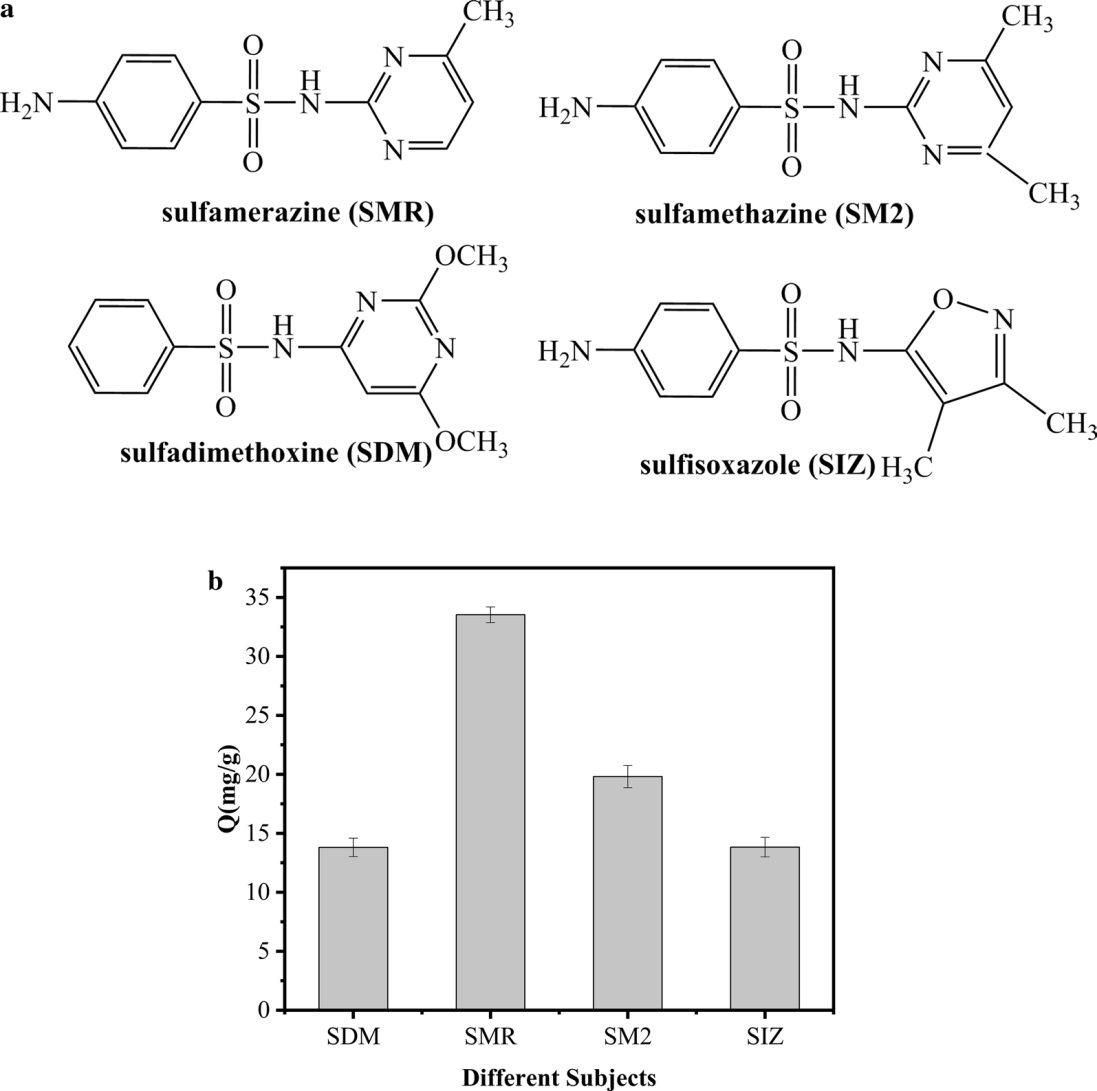
**a** Structures of the four sulfonamides. **b** Effect of substrate on adsorption capacity

Determination of selective adsorption: As shown in Fig. 7a, b, the SMR, SDM, SIZ and SM2 materials were similar in structure, but the adsorption capacity was much higher for SMR than for the other sulfonamides. We surmise that SMR is less sterically hindered than SM2, SDM, and SIZ, so is more easily adsorbed to the magnetic material [28].

**Fig. 8:**
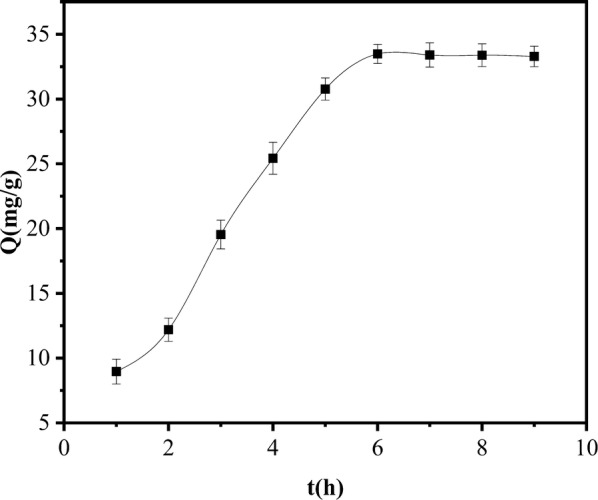
Temporal dynamics of SMR amount adsorbed to PSS magnetic material

Temporal changes in adsorption capacity: Fig. 8 plots the temporal changes in the amount of SMR adsorbed by the magnetic material. The initial SMR concentration was 0.6 mmol/L. The SMR was rapidly adsorbed during the first 5.5 h. After this time, the adsorption rate gradually decreased toward zero at adsorption equilibrium. The fast initial rate is attributable to the large number of adsorption active sites on the surface of the magnetic material. Over time, these sites gradually become occupied by SMR molecules, so the adsorption rate slows until adsorption equilibrium is reached. The adsorption time in subsequent experiments was thus chosen as 7.5 h.

Determination of number of reaction stages: Adsorption kinetics are commonly described by quasi-first-order and quasi-second-rate models. The quasi-first-order rate equation is also called the Lagergren first-order kinetic equation. The two models are usually linearized as [29].

3 $$\ln (Q_{e} - Q_{t} ) {\text{ = ln}}Q_{e} - {\text{k}}_{1} t$$

4 $${{\text{t}} \mathord{\left/ {\vphantom {{\text{t}} {Q_{\text{t}} }}} \right. \kern-0pt} {Q_{\text{t}} }}{\text{ = (k}}_{2} Q_{e}^{2} )^{{{ - }1}} { + }\;{{\text{t}} \mathord{\left/ {\vphantom {{\text{t}} {Q_{e} }}} \right. \kern-0pt} {Q_{e} }}$$

In these expressions, *Q*_*t*_ is the adsorption amount (mg/g) at time t, *Q*_*e*_ is the equilibrium adsorption amount (mg/g) of the material, and k_1_ (min^−1^) and k_2_ [g/(mg·min)] are the primary and secondary rate parameters, respectively.

Fitting the experimental data in Fig. 8 using quasi-first-order and quasi-second-order reaction kinetic equations,the results of the fitting are shown in Table 2. The reaction order was determined by the correlation coefficient of the regression equation and the difference between the experimental and computed *Q*_*e*_ values. The results show that the adsorption process of SMR by the PSS magnetic material was consistent with the second-order kinetic model. The adsorption quantity (Q_e_, cal) calculated by the second-order kinetic model is very close to the experimentally measured adsorption quantity (Q_e_, exp), and the correlation coefficient is good. In most cases, the Lagergren first-order kinetic equation can only be applied to the initial stage of the adsorption process rather than the entire stage; while the second-order reaction kinetic model assumes that the rate-limiting step may be chemisorption and is suitable for many adsorption studies [30].

**Table 2:** The results of kinetics analysis

Model	Initial SMR concentration (mmol/L)	Equations	K_1_ (min^−1^)	K_2_ (g mg^−1^ min^−1^)	*R*^2^	*Q*_*e*_,_cal_	*Q*_*e*,exp_
Pseudo first order kinetic model	0.6	ln(1 − *Q*_*t*_/*Q*_*e*_) = − 0.7013t + 0.7192	0.7013	–	0.7844	32.51 mg/g	33.53 mg/g
Pseudo second order kinetic model	0.6	t/*Q*_*t*_ = 0.0301t + 10.0866	–	0.00009	0.9998	33.22 mmol/g	33.53 mmol/g

**Fig. 9:**
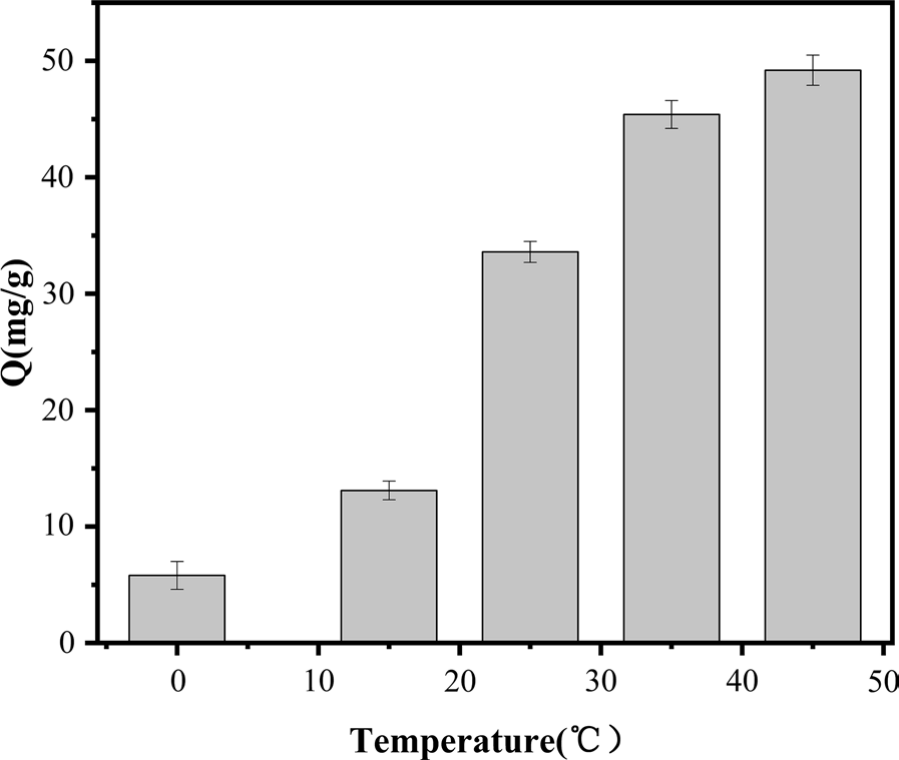
Effect of temperature on adsorption capacity of PSS magnetic material

Effect of temperature on adsorption capacity of the PSS magnetic material: Temperature is another important factor affecting the adsorption process. To assess the temperature dependence of SMR adsorption to the PSS magnetic material, the SMR concentration was maintained constant at 0.6 mmol/L (0.01 g magnetic material in 10 mL SMR–methanol solution), and the SMR content in the supernatant was determined after shaking for 7.5 h at 0 °C, 15 °C, 25 °C, 35 °C, or 45 °C in a constant-temperature oscillator. As shown in Fig. 9, the adsorption amount increased with increasing temperature, indicating that the adsorption process was endothermic.

**Fig. 10:**
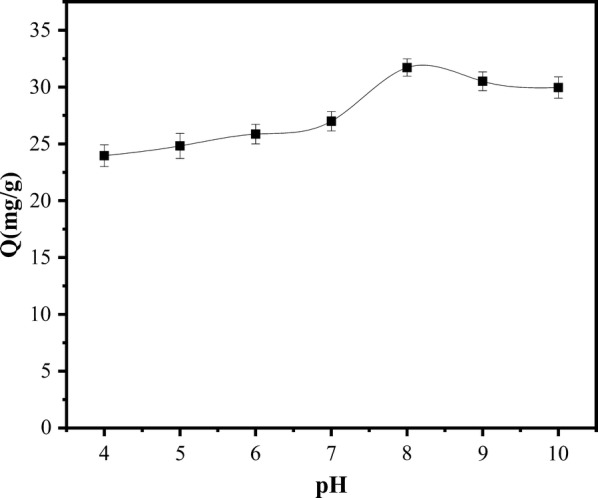
Effect of pH on adsorption capacity

Effect of pH on Adsorption Properties of Magnetic Materials: Fig. 10 shows the effect of pH on the adsorption properties of magnetic materials. It can be seen from the figure that the change of pH has different adsorption effects on its adsorption performance.

**Fig. 11:**
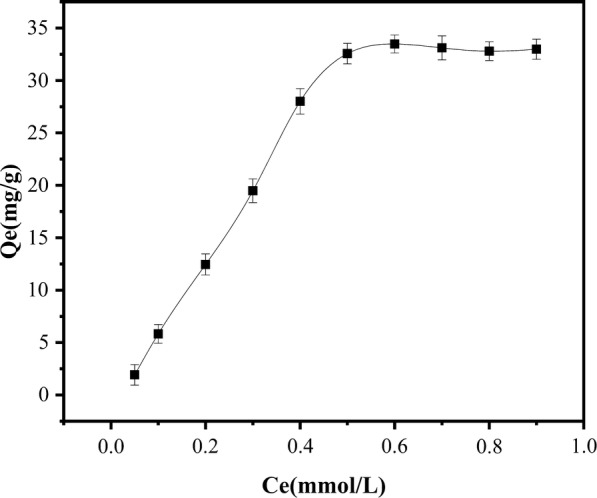
Adsorption isotherm of SMR at 25 °C

Adsorption isotherm model: The adsorption isotherm relates the equilibrium adsorption amount to the equilibrium concentration at a certain temperature. The equilibrium adsorption amount *Q*_*e*_ was calculated at various equilibrium concentrations *C*_*e*_ measured in the static equilibrium adsorption experiment. In this experiment, the adsorption isotherm of the magnetic material was obtained at 25 °C, and is plotted in Fig. 11.

As evidenced in Fig. 11, the amount of adsorbed SMR at equilibrium increased with equilibrium concentration. The saturated adsorption capacity of the magnetic material at 25 °C was 33.53 mg/g, higher than that of a molecularly imprinted polymer reported in the literature [31]. It appears that the magnetic material can have a higher adsorption capacity for the SMR. The above adsorption isotherm was then fitted by the Freundlich and Langmuir isotherm adsorption equations. The fitting parameters are shown in Tables 3 and 4, respectively. The correlation coefficients of both fits exceeded 0.95, and *n* in the Freundlich equation was greater than unity. It was concluded that both isotherm adsorption equations can adequately describe the adsorption process of SMR on magnetic materials.

**Table 3:** Fitting parameters of the Freundlich isotherm for SMR adsorption to PSS magnetic material

T (°C)	Fitting equation	*K*_*F*_ (mmol/g)	*n*	*R*^2^
25	ln*Q*_*e*_ = 0.2857ln*C*_*e*_ − 1.8923	0.1507	3.5002	0.9674

**Table 4:** Fitting parameters of the Langmuir isotherm for SMR adsorption to PSS magnetic material

T (°C)	Langmuir equation	*K*_*L*_ (L/mmol)	*Q*_*m*_ (mg/g)	*R*^2^
25	*C*_*e*_/*Q*_*e*_ = 0.0271*C*_*e*_+ 0.0018	15.5032	36.84	0.9836

Langmuir adsorption isotherm:

5 $${{C_{e} } \mathord{\left/ {\vphantom {{C_{e} } {Q_{e} }}} \right. \kern-0pt} {Q_{e} }} = {{C_{e} } \mathord{\left/ {\vphantom {{C_{e} } {Q_{m} }}} \right. \kern-0pt} {Q_{m} }} + \left( {K_{L} Q_{m} } \right)^{ - 1}$$

Freundlich adsorption isotherm:

6 $$\ln Q_{e} = n^{ - 1} C_{e} + \ln K_{F}$$

In Eq. (5), *Q*_*m*_ is the theoretical maximum adsorption amount (mg/g) of the material, and *K*_*L*_ is the Langmuir adsorption equilibrium constant (L/mg). In Eq. (6), *K*_*F*_ is the material adsorption capacity (mg/g), and *n* denotes the affinity of the material for the adsorbate. The results of the Langmuir and Freundlich isotherm adsorption equations are shown in Tables 3 and 4.

Calculation of thermodynamic constants: To investigate the thermodynamics of the adsorption process, we computed the Gibbs free energy change ΔG, the adsorption enthalpy change ΔH, and the entropy change ΔS [32]. The values of ΔG, ΔH, and ΔS are shown in Table 5. Judging from the negative Gibbs free energy, the SMR spontaneously adhered to the magnetic material under isothermal conditions. Meanwhile, the positive enthalpy indicates that the adsorption was an endothermic process, and that raising the temperature will enhance the adsorption. Physical and chemical adsorptions occur in different ΔH ranges: 2.1–20.9 kJ/mol and 20.9–418.4 kJ/mol, respectively [33]. The present results confirm that SMR adsorbed to the material surface by a chemical process. Moreover, the entropy change ΔS of the adsorption process was positive, indicating that SMR adsorption increased the degree of freedom of the liquid–solid interface.

**Table 5:** Thermodynamic parameters of adsorption

T (K)	ΔG (kJ/mol)	ΔH (kJ/mol)	ΔS [J/(mol K)]
288	− 2.394	38.29	141.26
298	− 2.478		136.81
308	− 2.561		132.63

### Testing in an actual food sample

**Fig. 12:**
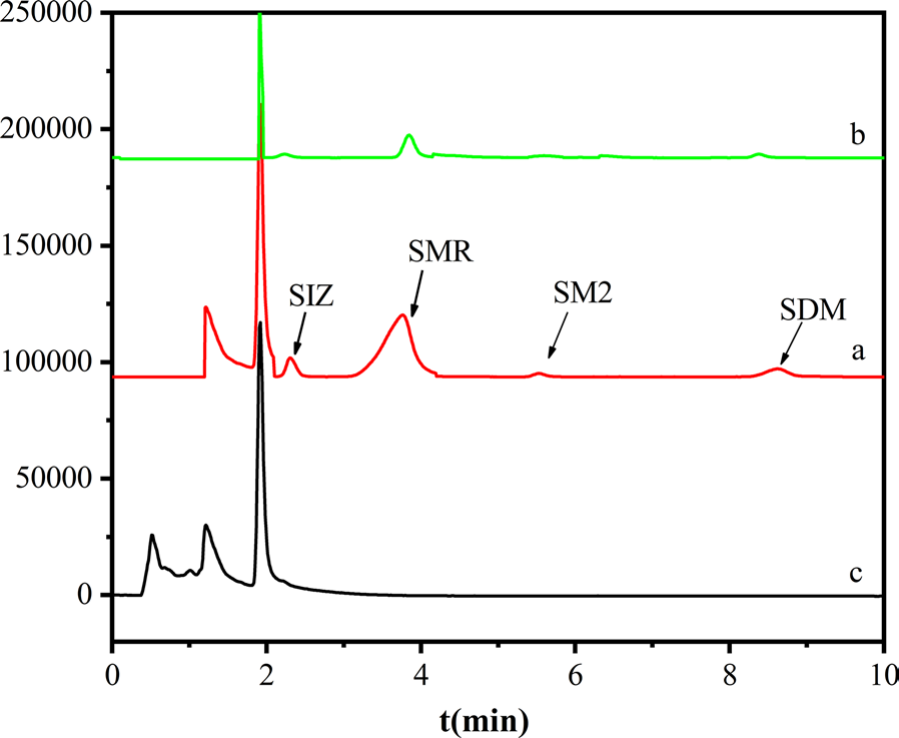
Adsorption chromatograms of SMR in milk samples: **a** SMR before adsorption by magnetic material **b**, after adsorption by magnetic material, and **c** blank sample

Finally, the milk sample treated with the PSS magnetic material was subjected to HPLC measurement, and as a result, as shown in Fig. 12c, no sulfonamide was detected. The adsorption chromatograms of SMR in the milk samples are shown in Fig. 12. No sulfonamide was detected in the blank sample (Fig. 12c). As the experimental samples, three kinds of sulfa antibiotics with concentrations of 10, 50 and 100 μg/mL were added to the milk samples by spiked recovery. After treatment, HPLC was performed. As shown in Fig. 12a, the four antibiotics SIZ, SMR, SM2, and SDM were well separated along the chromatogram, and were adsorbed by 0.10 g of magnetic material. At adsorption equilibrium, the particles were separated by applying a magnetic field, and the supernatant was extracted and subjected to HPLC measurements (Fig. 12b). The magnetic material effectively adsorbed the SMR from milk. The removal rates of SIZ, SMR, SM2, and SDM were calculated as 83.36%, 94.36%, 63.36%, and 58.41%, respectively, confirming that the magnetic material can adsorb and remove sulfa antibiotics from real liquid food samples.

Three SMR standard samples with concentrations of 10, 50 and 100 μg/mL were added by sample spike method. The experimental results are shown in Table 6. Different loading amounts were added to prepare different recovery rates and relative standard deviations. The recovery rate was 81.1 to 102.7%, and the relative standard deviation (RSD %) was 2.6 to 3.7%. The detection limit was 8.01 μg/L with a three-fold signal-to-noise ratio (S/N). It is proved that the magnetic material has a good adsorption detection study on SMR in milk.

**Table 6:** Recovery results of spiked SMR in milk (n = 3)

Sample	Scaling amount (μg/mL)	Recovery (%)	RSD (%)
SMR	10	81.1	3.7
	50	102.7	3.1
	100	98.3	2.6

## Conclusions

PSS magnetic material was prepared by the SI-ATRP technique. The adsorption properties, thermodynamics, and kinetic parameters of the material were investigated in the presence of sulfa antibiotics. SMR (the smallest molecular-weight sulfonamide) was selected for analysis. At 25 °C and an initial SMR concentration of 0.6 mmol/L, the saturated SMR adsorption capacity of the magnetic material was 33.53 mg/g. The adsorption properties of the sulfa antibiotics on the material were well fitted by the Langmuir and Freundlich equations. According to the thermodynamic parameters, The thermodynamic parameters indicate that the adsorption process is a spontaneous endothermic process, and the elevated temperature is favorable for adsorption. Kinetic studies show that the adsorption process conforms to the quasi-second-order kinetic equation.

## Data Availability

All data and material analyzed or generated during this investigation are included in this published article.

## Abbreviations

CAC: Codex Alimentarius Commission
NaSS: sodium styrene sulfonate
PSS: polystyrene sulfonate sodium
